# Short DNA Fragments Are a Hallmark of Heavy Charged-Particle Irradiation and May Underlie Their Greater Therapeutic Efficacy

**DOI:** 10.3389/fonc.2016.00130

**Published:** 2016-06-10

**Authors:** Dalong Pang, Sergey Chasovskikh, James E. Rodgers, Anatoly Dritschilo

**Affiliations:** ^1^Radiation Medicine, Georgetown University Medical Center, Washington, DC, USA; ^2^Radiation Oncology, Medstar Franklin Square Medical Center, Rosedale, MD, USA

**Keywords:** short DNA fragments, radiation, AFM, low-LET, charged particle

## Abstract

Growing interest in proton and heavy ion therapy has reinvigorated research into the fundamental biological mechanisms underlying the therapeutic efficacy of charged-particle radiation. To improve our understanding of the greater biological effectiveness of high-LET radiations, we have investigated DNA double-strand breaks (DSBs) following exposure of plasmid DNA to low-LET Co-60 gamma photon and electron irradiation and to high-LET Beryllium and Argon ions with atomic force microscopy. The sizes of DNA fragments following radiation exposure were individually measured to construct fragment size distributions from which the DSB per DNA molecule and DSB spatial distributions were derived. We report that heavy charged particles induce a significantly larger proportion of short DNA fragments in irradiated DNA molecules, reflecting densely and clustered damage patterns of high-LET energy depositions. We attribute the enhanced short DNA fragmentation following high-LET radiations as an important determinant of the observed, enhanced biological effectiveness of high-LET irradiations.

## Introduction

DNA is the critical target of ionizing radiation-induced cellular damage, and DNA double-strand breaks (DSBs) are the most lethal of more than 100 various DNA lesions induced by ionizing radiation (1–3). Biological observations implicate DNA DSBs resulting from high-LET radiation in cell death and carcinogenesis to a greater extent than that observed following low-LET radiations (4–6). Mechanisms underlying such observations have focused on dense and complex ionization events resulting in clustered DNA DSBs that are more difficult to repair (7, 8).

Established methods for measurements of DSBs include sucrose gradient sedimentation (9), neutral filter elusion (10), continuous or pulsed-field gel electrophoresis (PFGE) (11–13), the comet assay (14, 15), and, more recently, the γ-H2AX foci quantification (16, 17). DSBs induced in cellular environment and in denatured DNA have been determined (18–23); however, measured DSBs following high-LET radiations were reported equal to or only marginally greater than that observed following low-LET radiations (6, 24, 25). This is in contradiction to the observed greater relative biological effectiveness (RBE) by several fold for cell survival following high-LET radiation exposures (26, 27). However, a better correlation between RBE survival and DSB induction was found with assays of unrepaired DSBs (28–31).

This apparent discrepancy between RBE for cell survival as compared to DSB induction contradicted the accepted thesis of DSB as the primary lesion for cell killing. Subsequently, detailed examination of the techniques used for DSB measurements has revealed that they were reliable only for DNA fragments in the kilobase-pair region and possible shorter DNA fragments were potentially unaccounted for (32–34).

In addition to experimental investigation of DSB induction by ionizing radiation, theoretical modeling employing individual particle track structures has also been pursued (35–38). Ionizing events by individual particles based on established physics principles have shown that heavy charged-particle radiations produce a much greater clustered energy depositions (within a few base pairs) imparting sufficient energy to generate free radicals, which can lead to DNA DSBs or directly cause DSBs when occurring on the opposite strand within a certain distance (39–41). Such Monte Carlo simulations have revealed induction of short DNA fragments less than a few hundred base pairs by both low- and high-LET radiations, which were not quantified in experimental measurements (41, 42).

As a single molecule imaging instrument, the atomic force microscopy (AFM) offers the resolution to image individual atoms of solid state materials and nanometer resolution to visualize biological molecules, e.g., DNA molecules (43–46). Unlike Electron Microscopy or Scanning Tunneling Microscopy, AFM requires minimum sample preparation, reducing or eliminating potential distortions attributable to sample preparation (47, 48). In addition, its ability to measure biomolecules in aqueous solutions, similar to the native environment, offers the possibility for examining *in vitro* behaviors and interactions of biomolecules of interest (49–51).

We have previously reported the presence of short DNA fragments in neutron irradiated plasmid DNA, reflecting the high-LET energy deposition of neutrons (52). Here, we address the effectiveness of high-LET charged-particle irradiation in producing short DNA fragments in plasmid DNA. Use of plasmid DNA molecules as the targets allows for high-resolution imaging and easy identification of DNA fragmentation in sizes of a few to a few hundred nanometers in lengths. We investigated DNA fragmentation following radiations of the low-LET Co-60 photon and electron, and the high-LET Beryllium and Argon ions.

## Materials and Methods

### DNA Samples

Plasmid DNA (pUC19, 2686 bp in length) was purchased from New England Biolab at a concentration of 1000 μg/ml in HEPES buffer (Beverley, MA, USA). The samples were diluted to a concentration of 5 μg/ml in buffer containing 10 mM HEPES and 1 mM MgCl_2_ and aliquoted into vials containing 250 μl DNA solution each.

### Irradiation

Irradiation of the aliquots of DNA solutions was performed at the following sites.

Electron irradiations were performed at the Georgetown University Medical Center in Washington, DC, USA on a medical linear accelerator with 6 MV energy (Varian 2100 C/D, Varian, Palo Alto, CA, USA) to doses of 1000–8000 Gy in 1000 Gy increment. The dose was calibrated using a NIST traceable ionization chamber.

Co-60 photon irradiations were performed at Neutron Products in Dickerson, MD, USA using an industrial Co-60 irradiator at a dose rate of 20 kGy/h in the same dose range as that for electrons.

Beryllium ion irradiations were performed at the Oak Ridge National Laboratory in Oak Ridge, TN, USA. The energy of the Beryllium particle beam was 100 MeV/n, and the LET was 11.6 keV/μm. The doses delivered ranged from 3 to 12 kGy, calculated as the product of the particle fluence rate and the LET of the ion multiplied by the time the beam was on.

Argon ion irradiations were performed on the HIMAC charged-particle accelerator at the National Institute of Radiological Science in Chiba, Japan. The energy of the Argon ion beam was 390 MeV/n, and the LET was 99.5 keV/μm. The doses delivered were 3–12 kGy, using a similar way for dose determination as that for Beryllium irradiation.

As a control, a set of three un-irradiated DNA samples was prepared for each experiment.

### AFM Imaging

A Bruker Nano Scope IIIa AFM (Bruker, Santa Barbara, CA, USA) was used for DNA imaging in tapping mode in air. The AFM cantilevers were commercially available from Bruker with a tip radius of approximately 10 nm. Sample preparation for imaging consisted of deposition of 2 μl of the DNA solution on freshly cleaved mica surface, followed by a gentle rinse with 1 ml of distilled water and subsequent drying in the gentle flow of Nitrogen gas. The Scanning frequency was 1 Hz and typical scanning size was 2 μm × 2 μm.

The sizes of the DNA fragments in each image were measured individually using the NanoScope IIIa software. Over a thousand DNA fragments were measured for each irradiated DNA sample to ensure a statistical uncertainty of <5%. Fragment size distribution profiles relating the numbers of DNA fragments to their sizes were constructed. The average numbers of DSBs per DNA, per broken DNA, and DSB distributions as a function of spatial distance were derived from the constructed size distribution profiles. For details on the technique and data analysis, the reader is referred to our previous paper (52).

## Results

**Figure 1:**
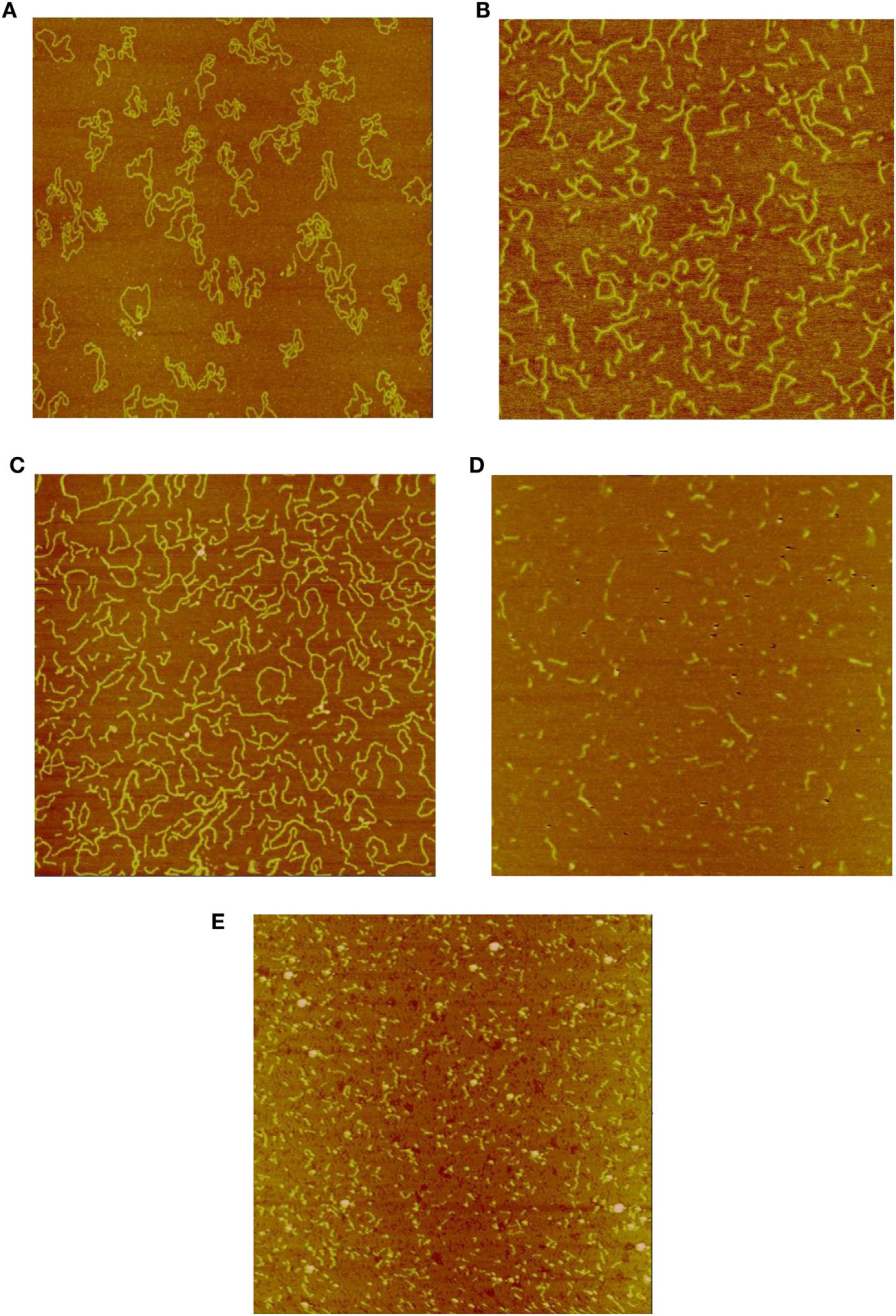
**(A)** Sample image of un-irradiated pUC19 plasmid DNA. The size of the image is 2 μm × 2 μm, as that for the rest of the images. **(B)** Sample image of Co-60 photon irradiated pUC19 plasmid DNA. The radiation dose is 6 kGy. **(C)** Sample image of electron irradiated pUC19 plasmid DNA. The radiation dose is 6 kGy. **(D)** Sample image of Beryllium ion irradiated pUC19 plasmid DNA. The radiation dose is 6 kGy. **(E)** Sample image of Argon ion irradiated pUC19 plasmid DNA. The radiation dose is 6 kGy.

Figures 1A–E show representative AFM images of the plasmid DNA of un-irradiated controls and following irradiation by Co-60 photon, electron, Beryllium, and Argon ions to doses of 6 kGy. As shown in Figure 1A, the majority of the control DNA molecules were in relaxed circular conformation with occasional super coiling of one or two twists. In Figures 1B,C, the amount of DNA fragmentation and sizes appear similar, demonstrating similar physical characteristics of low-LET energy deposition patterns following Co-60 photon and electron irradiations. Examination of Figures 1D,E shows that DNA fragmentation is markedly greater than that shown in Figures 1B,C. Furthermore, the average sizes of DNA fragments are shorter, demonstrating the enhanced capability of the high-LET Beryllium and Argon ions to fragment DNA to a much greater extent.

**Figure 2:**
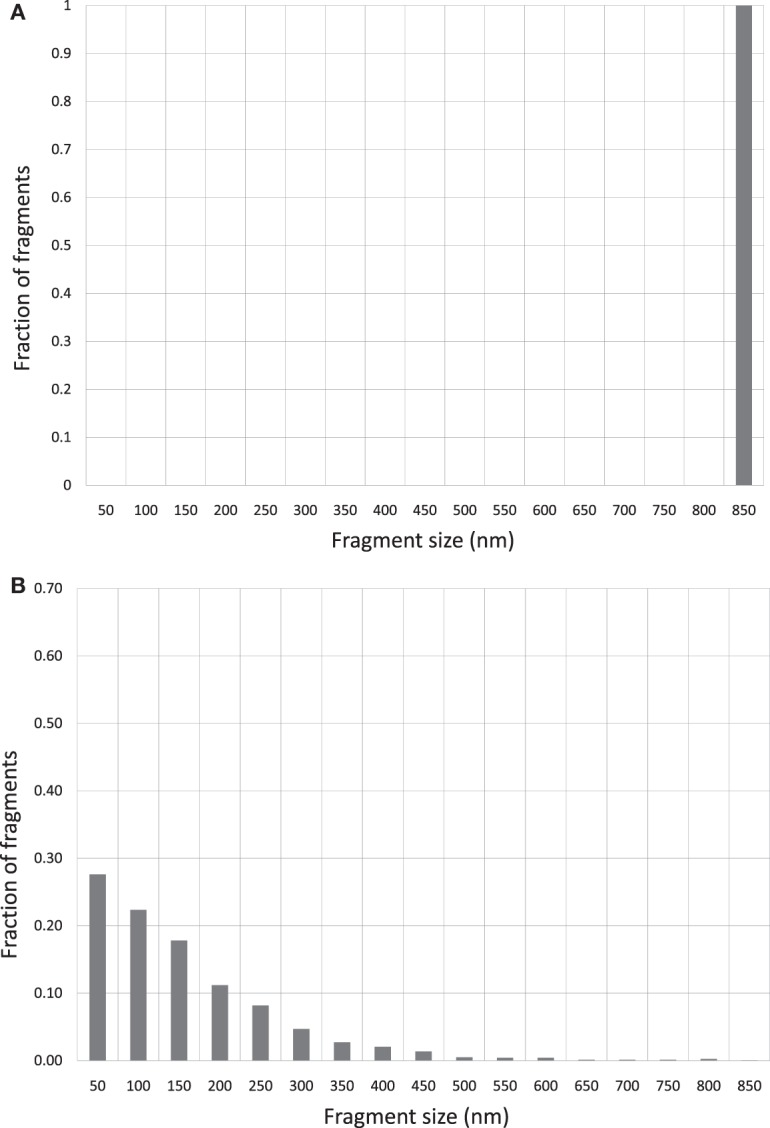

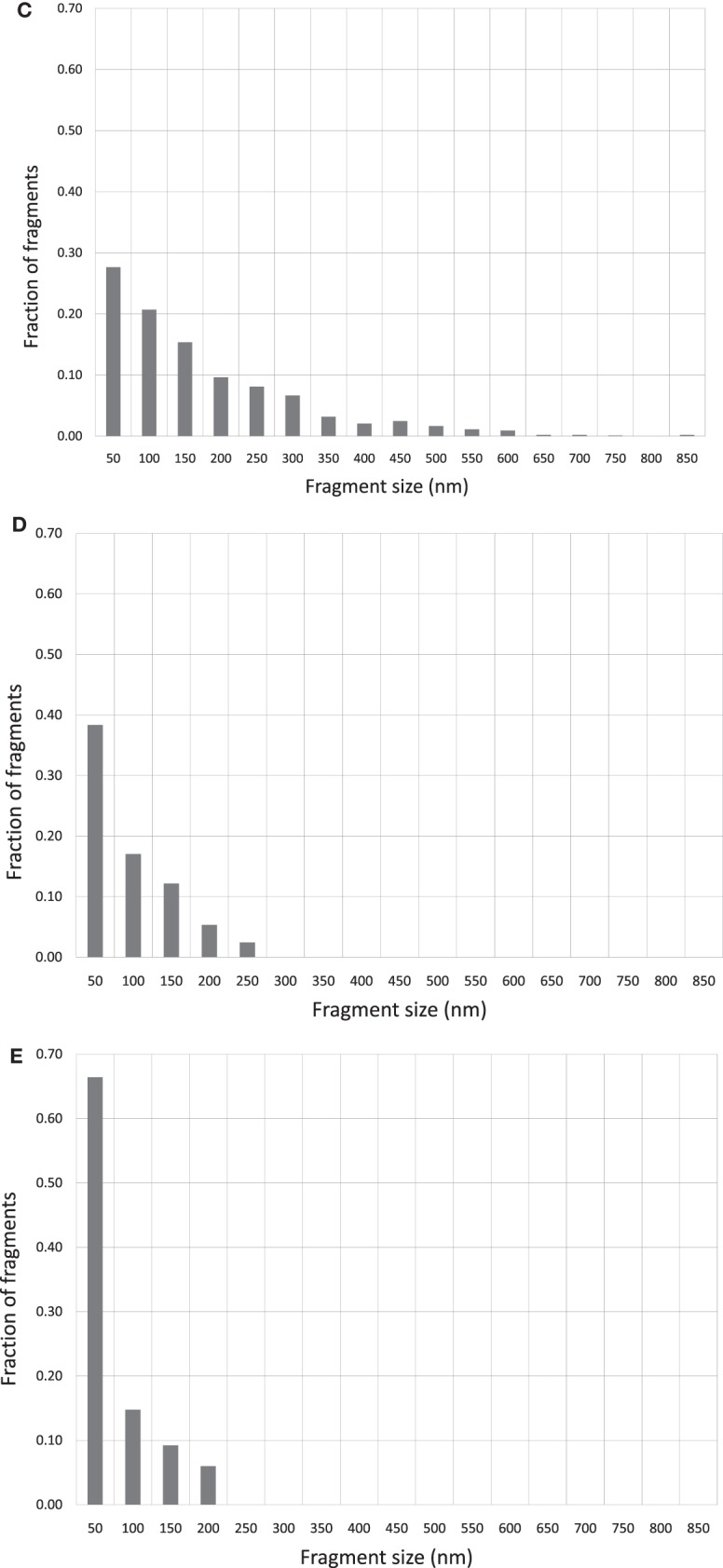
**(A)** DNA fragment size distribution of un-irradiated pUC19 plasmid DNA. **(B)** DNA fragment size distribution of 6 kGy Co-60 photon irradiated DNA. **(C)** DNA fragment size distribution of 6 kGy electron irradiated DNA. **(D)** DNA fragment size distribution of 6 kGy Beryllium ion irradiated DNA. **(E)** DNA fragment size distribution of 6 kGy Argon ion irradiated DNA.

Figures 2A–E show the corresponding reconstructed DNA fragment size distributions based on individually measured DNA fragment sizes for each irradiated samples. The size of the original, un-fragmented pUC19 plasmid DNA is 850 nm and is evenly divided into 50 nm bins in the range of 0–850 nm. Size profile of the un-irradiated DNA was marked by a near 100% uni-spike at the 850 nm bin, represented by the unbroken and occasional DNA molecules with one break only. Mirroring images shown in Figures 1B,C, the DNA fragment size distributions in Figures 2B,C are essentially identical, and approximating an exponential distribution as a function of the fragment sizes. However, the size distributions shown in Figures 2D,E are quite different from that in Figures 2B,C, marked by pronounced spikes of fragments in the shortest bin of 50 nm. This demonstrates a much enhanced induction of short DNA fragments by the Beryllium and Argon ions. Size distributions in bins longer than 50 nm follow a similar exponential-like distribution as that in Figures 2B,C, but at a more accelerated drop off with increasing fragment size.

Based on the measured DNA fragment sizes, the average numbers of DNA DSB per DNA molecule are derived for DNA molecules including both fragmented and intact DNA. In addition, DSBs per DNA for fragmented DNA molecules only are also derived to further illustrate the DNA fragmentation capability by different types of radiation. Derivation of these quantities is based on the following considerations. If a plasmid DNA contains only one DSB, it becomes linearized as a single linear DNA fragment of the original length of 850 nm; if it contains two DSBs, a plasmid is broken into two pieces and the combined lengths of the two fragments add up to the original DNA length and this pattern holds for DNA containing N DSBs. Therefore, the number of fragments equals the number of DSBs, and consequently, the number of DSBs per DNA molecule simply equals to the number of fragments divided by the total number of DNA molecules from which the fragments are originated, which can be calculated as the sum of all the fragment lengths divided by the length of an intact DNA.

**Figure 3:**
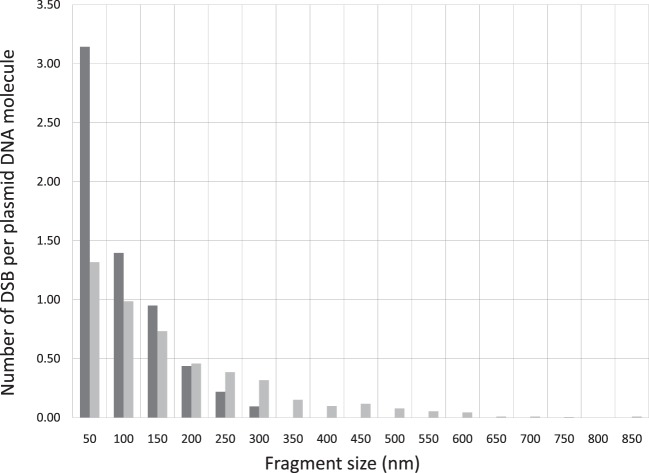
**DSB spatial distribution on a pUC19 plasmid DNA molecule induced by Beryllium ion and electron irradiation to a dose of 6 kGy**.

In addition to the average number of DSB per DNA, which provides a general indication of the DNA breaking capability by ionizing radiation, information on the spatial correlation of the DSBs on a DNA molecule can be further derived from the size distributions. As an illustrative example, we calculate the number of DSBs distributed within a distance of 50 nm on a DNA molecule. This is derived by counting the number of fragments in the length interval from 0 to 50 nm, which is then divided by the total number of DNA molecules as determined in the previous paragraph. This calculation can be extended to determine DSBs distributed in other longer length intervals. By this information, we obtain a clear indication of whether DSBS are distributed in a confined small spatial region or more spread out on a DNA molecule. Correlation of this DSB distribution pattern with the type of radiation provides a simple measure for the assessment of ionization clustering. In Figure 3, we construct the number of DSBs per DNA for electron and Beryllium irradiated DNA samples to a dose of 6 kGy in relation to the fragment sizes to demonstrate the DSB spatial distribution on a DNA molecule. Clearly, Beryllium ions induce more dense and localized DSBs, whereas electrons generate more uniformly distributed DSBs on a DNA molecule, demonstrating the high degree of DNA damage clustering by high-LET irradiations.

We further calculated the RBE for DSB induction as a function of radiation quality. The RBE calculated in this report is defined as the ratio of the number of DSBs per unit DNA molecule of a given type of radiation to that by Co-60 photon. Table 1 gives the DSB per DNA molecule for the radiations investigated and the corresponding RBEs determined at 6 kGy. For comparison purposes, we also have included the RBE for neutron studied in a previous publication (52).

**Table 1:** **Measured DSB per DNA molecule and corresponding RBE for radiations investigated in this and a previous report (52)**.

Radiation	DSB/DNA	STD	RBE	STD	LET (keV/um)
Electron	4.77	0.4	0.82	0.08	0.2
Co-60	5.83	0.33	1.00	0.08	0.2
Neutron	7.36	0.78	1.26	0.15	55
Be	6.24	0.71	1.07	0.14	11.6
Argon	26.09	5.69	4.48	1.01	99.5

*The comparison was made for the radiation dose of 6 kGy. RBE was calculated with Co-60 as the reference. The LET value for neutron is the average LET of the recoil protons generated by the primary neutrons, and the LET for Co-60 photon is the average LET of the secondary electrons produced by the primary photon through Compton effects*.

## Discussion

In this report, we employ AFM for the measurement of DNA fragmentation by the charged particles of Beryllium and Argon in comparison to that by the low-LET photon and electron to demonstrate the enhanced DNA fragmentation capability of high-LET radiations. As shown in the AFM images, short DNA fragments are produced after plasmid DNA exposure to both low- and high-LET radiations. However, the relative amounts of short DNA fragments are substantially greater after high-LET irradiations, with Beryllium and Argon ions, demonstrating a prevalence of clustered DNA DSBs produced by high-LET radiations not previously quantified due to limitations in the conventional biological techniques.

As discussed in the Section “Introduction,” the RBE for cell killing reported in the literature are generally a few fold higher for high-LET radiations (4, 5), but the DSB induction as measured using gel electrophoresis or other biological techniques are approximately unity or only slightly higher (25), presenting a contradiction to the fundamental concept of lethality of DSBs. Using Monte Carlo modeling of radiation-induced DNA damage, the groups led by Paretzke and Goodhead have reported clustered DNA lesions after exposure of modeled DNA molecules to high-LET radiations (38, 41, 53, 54). Campa and coauthors have further calculated the frequency of short DNA fragments generation by high energy protons and ions (42). The prominence of short DNA fragments induced by high-LET radiations presented in this and our previous publications, as well as reports by other investigators, provide experimental validation of the model-predicted short DNA fragments (52, 55, 56). It is apparent that short DNA fragments were undetected by techniques exploiting the migratory property of DNA fragments in gels, leaving accounted the DSBs corresponding to short DNA fragments, in particular, DSBs induced by high-LET radiations. It appears likely that with short DNA fragments included, the DSB induction by high-LET radiations should correlate better to RBE for cell survival.

To evaluate the capacity for DNA strand breakage by radiations of different quality, we calculated the RBE for DSB induction by the radiations investigated in this report together with that by neutrons for which the DNA fragment size distributions have been reported before (52). As shown in Table 1, the RBE increases as the LET of radiation increases, demonstrating the greater capacity of DNA damage by high-LET radiations. The ability of AFM to image short DNA fragments has permitted measurement of DSBs produced in close proximity resulting from clustered DSBs by high-LET radiations, and therefore offers a sensitive technique to quantify clustered DSBs not easily measurable using conventional biological methods.

In a previous report, we investigated the biological significance of short DNA fragments in DNA damage and repair and their potentially important roles in cell survival and carcinogenesis (57). We evaluated DNA binding and rejoining by Ku and DNA-PK, two major DNA repair proteins involved in the non-homologous end-joining (NHEJ) pathway and confirmed reports by other investigators on the minimum DNA length requirements for protein binding and activation (58, 59). When DNA fragments are short, the challenge to rejoin and repair them by the cell’s repair mechanisms becomes greater. Furthermore, the presence of un-rejoined and repaired short DNA fragments in cells can trigger genomic instability, leading to mutation or cell death by way of apoptosis (57, 60). Compared to longer DNA fragments, which are more frequently produced by low-LET radiations, short DNA fragments present a more lethal challenge to cellular repair mechanisms and survivability after exposure to high-LET radiations.

The fragment size distribution data for Co-60 photon and Argon ion at 10 kGy presented in this paper were for illustrative purpose only to show the greater capacity of high-LET radiations in generating short fragments. That data, as well as the data at 6 kGy presented in this report, are a subset of the range explored in our experiments. Naturally, it would be desirable to construct a complete dose–response for DSB induction for all the doses and radiation types investigated. However, contamination of certain samples has precluded AFM image acquisition of sufficient quality for more extensive analysis as we performed in our previous study of neutron and electron irradiations (55). Nonetheless, the DSB data at 6 kGy clearly show a radiation quality dependence of RBE for DSB induction.

The RBE for DSB induction has been measured for both cells and in aqueous solutions. Prise et al. summarized the DSB induction data for radiations of varying quality for various cell line (25). It was shown that the RBE generally remained approximately close to 1 for a wide range of LET values from 10.9 to 998 keV/μm. In a subsequent report, Prise et al. presented additional DSB induction data for a few additional ions in the LET range of 40–225 keV/μm obtained with the PFGE either using the fraction of activity released (FAR) or fragmentation method and showed a substantial difference in RBE values obtained with these two techniques (61). Again, the RBE values obtained with the FAR method remained close to 1 or less, but varied from 1.1 to 1.5 when measured using the fragmentation method. They concluded that the fragmentation method permitted quantification of shorter DNA fragments that were not measured with the FAR method and thus resulted in increased DSB collection.

The RBE values determined in this report were based on AFM measurement of individual DNA fragments induced in aqueous solution that were orders of magnitude shorter than what measured using the gel electrophoresis fragmentation method. The much larger RBE values obtained here reflect a much enhanced capability of AFM to measure short DNA fragments. It is, however, difficult to make a direct comparison of these RBE values to what Prise and coauthors have summarized, as our DNA model system is plasmid DNA in aqueous solution, while that in Prise’s report were DNA in cellular environments. The different DNA configuration and the substantially greater scavenging capacity of cells influence greatly the induction of DSB. Nevertheless, the techniques employed for DSB measurement have much greater impact on the accuracy of RBE values determined.

Therapeutic application of proton and heavy charged-particle irradiation has been gaining increasing acceptance, recognition, and popularity in the radiation oncology community worldwide (62–64). Heavy charged particles possess highly desired dosimetric advantages over photon or electron irradiations, exemplified by their finite range in tissue and Bragg peak in energy deposition (65). Furthermore, the biological advantage, as represented by their greater RBE for cell survival, adds another important dimension to the medical application of charged-particle irradiation. As presented in this and previous studies, heavy charged-particle radiations produce significantly more short DNA fragments than do low-LET radiations. We propose that the greater RBE of high-LET radiations is a result of the increased production of short DNA fragments by high-LET radiations.

## Conclusion

Atomic force microscopy imaging of plasmid DNA molecules as the DNA targets of irradiation demonstrates that heavy charged particles induce a significantly greater proportion of short DNA fragments than observed following low-LET irradiations. The increased short DNA fragment generation is attributed to clustered DNA DSB generation following high-LET irradiations. The increased short DNA fragment production may be a critical factor underlying the greater biological effectiveness of heavy charged-particle radiation.

## Author Contributions

DP designed, performed the experiments, data analyses, and wrote the manuscript; SC performed AFM imaging of the DNA samples; JR participated in electron and Co-60 irradiation experiments and reviewed the manuscript; AD participated in design of the experiments, reviewed, and edited the manuscript.

## Conflict of Interest Statement

The authors declare that the research was conducted in the absence of any commercial or financial relationships that could be construed as a potential conflict of interest.
